# NURSING HOME SOCIAL SERVICES DIRECTORS' ROLE AND RESPONSIBILITIES

**DOI:** 10.1093/geroni/igac059.1564

**Published:** 2022-12-20

**Authors:** Mercedes Bern-Klug, Colleen Galambos

## Abstract

While most of the nursing home literature focuses on residents and nurses, there is a growing literature on social services staff and family members of nursing home residents. Social services staff include social workers as well as non-social workers with responsibility for assessing and care planning for resident psychosocial issues, as well as a host of other responsibilities including working with families. This symposium presents findings from the National Nursing Home Social Services Directors Survey (NNHSSDS), a cross-sectional (2019) nationally representative study (n=924) that collected data on social services staff characteristics, departmental role responsibilities, and training needs. In this session three papers developed from these data are presented to document the extent to which social services staff are involved with family members, their interest in receiving training on how they can support other staff in understanding resident abuse and exploitation (at the hands of family, fellow residents, or staff), and neglect. As demanding as the social services role is, many holding the position of social services director report they are thriving (not just surviving) in the role. A final paper describes which variables help to distinguish those who report they are thriving versus those who do not. Implications to be discussed include what administrators could do to make the social services director’s job more attractive. The RRF Foundation for Aging provided data collection support for the NNHSSDS.

